# H-SPAR DB: human spaceflight platform for analysis and research—an integrative omics database for space health

**DOI:** 10.1093/database/baaf083

**Published:** 2026-01-15

**Authors:** Marios Tomazou, Marilena M Bourdakou, Eleni Nicolaidou, Grigoris Georgiou, Kyriaki Savva, Efi Athieniti, Styliana Menelaou, Sotiroula Afxenti, George M Spyrou

**Affiliations:** Bioinformatics Department, The Cyprus Institute of Neurology and Genetics, 2371 Nicosia, Cyprus; Bioinformatics Department, The Cyprus Institute of Neurology and Genetics, 2371 Nicosia, Cyprus; Bioinformatics Department, The Cyprus Institute of Neurology and Genetics, 2371 Nicosia, Cyprus; Bioinformatics Department, The Cyprus Institute of Neurology and Genetics, 2371 Nicosia, Cyprus; Bioinformatics Department, The Cyprus Institute of Neurology and Genetics, 2371 Nicosia, Cyprus; Bioinformatics Department, The Cyprus Institute of Neurology and Genetics, 2371 Nicosia, Cyprus; Bioinformatics Department, The Cyprus Institute of Neurology and Genetics, 2371 Nicosia, Cyprus; Bioinformatics Department, The Cyprus Institute of Neurology and Genetics, 2371 Nicosia, Cyprus; Bioinformatics Department, The Cyprus Institute of Neurology and Genetics, 2371 Nicosia, Cyprus

## Abstract

H-SPAR DB is a comprehensive database designed to support space health research by providing a unified platform for data integration, analysis, and interpretation. The database simplifies the complex workflows associated with spaceflight-related biology studies by combining curated molecular lists, transcriptomic datasets from NASA’s GeneLab, and user-uploaded data into a streamlined, user-friendly interface. H-SPAR DB enables researchers to perform differential expression analysis, set operations, and association analyses while also generating integrative knowledge graphs around a space-related biological theme. The platform reduces the time required for data gathering and processing by offering a single platform for data exploration, analysis, and visualization. By integrating interactive visualizations and data tables, H-SPAR DB facilitates the interpretation of results, ultimately enhancing the efficiency of space biology research and fostering discoveries that address human health challenges in space. Researchers can access H-SPAR DB freely at https://bioinformatics.cing.ac.cy/H-SPARDB/ without login or other requirements.

## Graphical Abstract

**Graphical Abstract:**
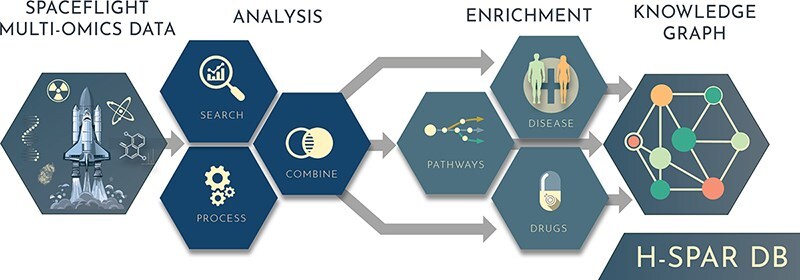

## Introduction

Space exploration represents one of humanity’s most ambitious and rewarding endeavours. As we venture further into the cosmos, a crucial challenge lies in understanding at the biomolecular level the health impacts of spaceflight on human physiology, particularly during extended missions [1]. The unique stressors of space, including microgravity, radiation, and confinement, induce significant physiological changes at the molecular level, potentially leading to detrimental effects like muscle atrophy, bone loss, immune dysfunction, and cardiovascular alterations [2]. While resources like NASA’s GeneLab (https://osdr.nasa.gov/bio/repo/) [3] offer valuable omics data, the complexity of analysing and interpreting these datasets presents a significant hurdle, especially for researchers lacking bioinformatics expertise. This gap hinders the full potential of GeneLab and slows the pace of discovery in space-related health research.

Moreover, much space-related biological data exist outside GeneLab, scattered across various publications and databases. In recent years, new international efforts have emerged to address this challenge through large-scale, integrated space biology resources. The Space Omics and Medical Atlas (SOMA) represents a landmark initiative providing harmonized, multi-omic, clinical, and cellular datasets from astronauts across multiple space missions, enabling systems-level comparisons and cross-mission integration [4]. In parallel, recent studies have demonstrated the power of integrative omics to uncover coordinated molecular responses to spaceflight, highlighting the importance of multi-layer biological data in understanding astronaut health [1, 5]. Complementing these efforts, recent work in the emerging field of astroimmunology has provided multi-omic insights into how spaceflight stressors disrupt immune regulation and host–microbiome interactions, revealing new biomedical challenges and countermeasures for future deep-space missions [6].

To fully leverage this information, a centralized repository is needed to consolidate these datasets, enabling collaboration, data sharing, and integrative analyses, with an option for users to upload their own data. Advanced analytical tools, such as association analysis, are essential to identify relationships between genes, pathways, diseases, and drugs, pinpointing molecular mechanisms affected by space conditions.

H-SPAR DB provides a comprehensive database designed to address these challenges by offering an interactive, user-friendly platform that integrates diverse space-related omics datasets with robust bioinformatics tools. Through its seamless interface, users can perform differential expression analysis, association studies, and generation of a knowledge graph as a network of interconnected diseases, molecular pathways, drugs, and other ontologies, enabling a holistic approach to space health research. By reducing the barriers to complex bioinformatics workflows, H-SPAR DB fosters collaboration and accelerates discoveries that are essential for human space exploration.

## Materials and methods

### Implementation

The core of H-SPAR DB is built using R (v4.4.2), Shiny (v2.3.4), and JavaScript, which provides interactive web-based functionalities for data analysis and visualization. The backend leverages several R packages for differential expression analysis, visualizations as well as network construction and analysis. The full list of packages is listed under the Help page of the database. H-SPAR DB is compatible with all major operating systems and modern browsers and is completely free and open to all users, with no login required.

### Design, workflow, and data sources of H-SPAR DB

H-SPAR DB is structured into two primary sections—Datasets and Integrative Analysis—which are accessible through the main sidebar of the database. The main workflow concept is to first allow the user to find, generate, filter, and combine gene sets of interest from various sources, which are then forwarded for association analysis against numerous enrichment libraries, which can be finally combined and analysed under a knowledge graph context. Specifically, the *Dataset modules* include:

Mined data: The module includes manually curated molecular sets from spaceflight-related studies in peer-reviewed PubMed-indexed literature (https://pubmed.ncbi.nlm.nih.gov/). To ensure thorough coverage, we employed a broad range of space-related keywords in our search strategy (Supplementary File ST1, keywords and PubMed queries used to identify space-related studies). After curation, we identified 461 relevant publications, yielding a dataset of 3798 genes, 1630 proteins, 61 non-coding RNAs, 50 metabolites, and 16 chemical compounds. Proteins are mapped to their genes using the UniProt.ws R package [7], microRNAs to their validated gene targets with multiMiR [8], and all gene symbols to their Ensembl, Entrez, and HGNC IDs using biomaRt [9, 10].
Differential expression: The module allows the user to perform on-the-fly RNA sequencing (RNA-seq) and microarray differential expression analysis using simple uni- or multivariate study designs, leveraging datasets sourced from NASA’s GeneLab (Supplementary File ST2, summary of transcriptomic datasets from GeneLab). The user can select datasets that contain the space-related stressor or other factors, construct the groups for the comparisons, and obtain rich visualizations and tables of the results. Differential expression analysis is conducted using DESeq2 R package (v1.46.0) [11] for count RNA-seq data and LIMMA R package (v3.62.2) [12] for normalized microarray intensities—both widely accepted methods for differential expression analysis.
User upload: The database allows the user to upload custom gene sets of interest with minimal effort in the simplified format of a four-column .csv file.

The *Integrative Analysis section* features downstream analysis modules:

Selected sets: All gene sets of interest identified and stored from the Datasets modules are summarized under this module. The user can perform basic set operations such as intersection, union, or subtraction to further refine or combine the sets of interest.
Association analysis: The module leverages enrichment databases to associate the selected gene sets to diseases, molecular pathways, candidate drug etc., using the Enrichr R package (v3.4) [13]. The database supports 22 enrichment libraries including GO [14], KEGG [15], DisGeNET [16], HPO [17], and others (Supplementary File ST3, selected libraries for association analysis) with approximately 38,000 enrichment terms in total.
Integrative knowledge graph: Finally, the Integrative Knowledge Graph module generates and visualizes networks derived from the selected biological terms in the previous step. Initially, it generates a reference or background network, where nodes represent all associated terms across the selected sets, while edges indicate common genes. The network generation covers horizontally heterogeneous enrichment libraries such that biological pathways can be connected to phenotypes, diseases, candidate drugs, and other ontologies, offering a systems-level understanding of molecular relationships. For each enrichment set, from each respective gene set, the module generates subnetworks aimed at highlighting which terms (nodes) are highly affected under different conditions. Subnetworks represent smaller, focused sections of the overall network, enabling users to visualize and interpret how specific biological terms or pathways are interrelated within a larger system. Users can compare the subnetworks and background pairwise, get detailed network metrics, edge, and nodal context, and finally export their project as individual tables or as an R (.rds) object.

### Output and visualization

H-SPAR DB allows users to explore their analysis results through detailed tables and interactive visualizations. For Mined Data, retrieved results are displayed in an interactive table, allowing users to filter data based on their research question. Results can also be visualized as interactive networks in the Analytics View section. Each analytical panel can be expanded to full-screen mode, allowing users to explore interactive network visualizations of the identified data sources. These options, along with detailed instructions, are described in the online Help page under the *Features and Workflows* section. The DE Results tab provides multiple outputs for in-depth analysis. The top section includes raw and normalized counts, the fitted Biological Coefficient of Variation (BCV) model, and a distance matrix heatmap. Differentially expressed genes (DEGs) are visualized in a volcano plot and listed in an interactive table, where users can click on a point in the plot to examine specific gene comparisons. In the Selected Sets tab, results are presented in interactive tables, and set operations are visualized using an UpSet plot. The UpSet plot provides a graphical representation of overlapping and unique elements among multiple gene sets, serving as an alternative to Venn diagrams and allowing users to easily identify shared or distinct DEGs across experimental conditions. The Association Analysis tab displays results through dot plots, dendrograms, and interactive tables, helping users interpret functional enrichments.

All interactive data tables that are dynamically generated and filtered in the user interface (UI) can be downloaded in excel, .csv, or .pdf format using the ‘Download’ button at the top of each table. All tables can be filtered using their search fields or through automated filtering functions implemented in the platform in order to enhance the user’s experience. The database offers access to large and complex public datasets. To allow effective navigation and interpretation of the results, the visualizations generated in the platform are interactive with reference to the users click, hover, or drag actions. Each action triggers specific feedback. For example, a click on the main barplot dives in the sourced literature, a hover over a knowledge graph node indicates its name and corresponding connectivity metrics while a selection of points in the differential expression volcano plot, shows the corresponding group-comparison boxplot. The database offers interactive tours for familiarizing the user with its interface, along with simplified example loading options and a thorough Help page.

## Results

### Use case scenario 1: uncovering radiation-induced molecular mechanisms with H-SPAR DB

**Figure 1.**
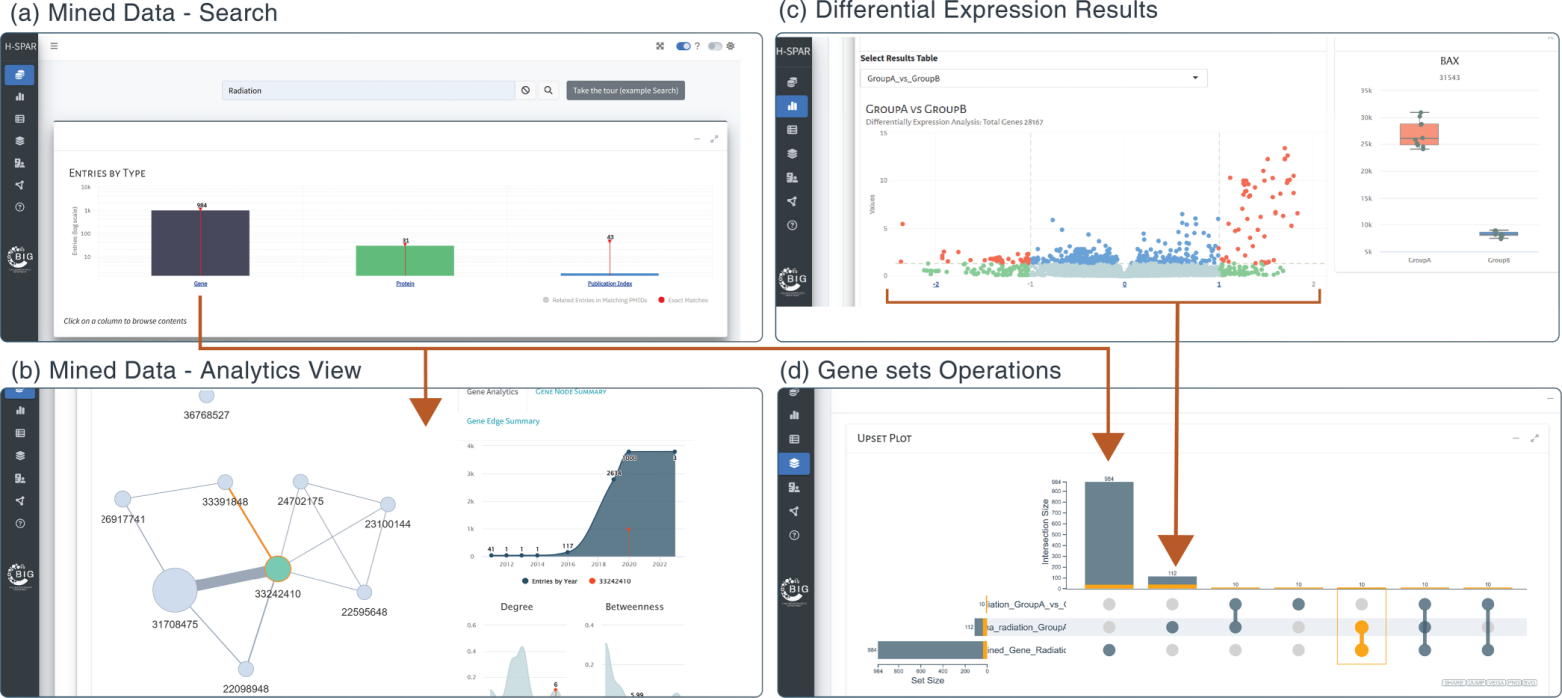
Application screenshots for mined data search, differential expression analysis, and set operations. (a) The main search field in the Mined Data tab allows the user to filter entries using specific keywords (e.g. ‘Radiation’) and summarize the results in the interactive barplot. (b) Expanding the analytics view box under each data type table (e.g. Genes), the user can access a network view of the search results along with various analytics. (c) Interactive volcano plot showing the differential expression analysis results following group comparisons. (d) The sets of genes selected from searching the mined data entries in panel (a) and significantly differentially expressed genes in panel (c) are forwarded as sets which are visualized as an interactive upset plot showing individual sets and all pairwise intersections.

**Figure 2.**
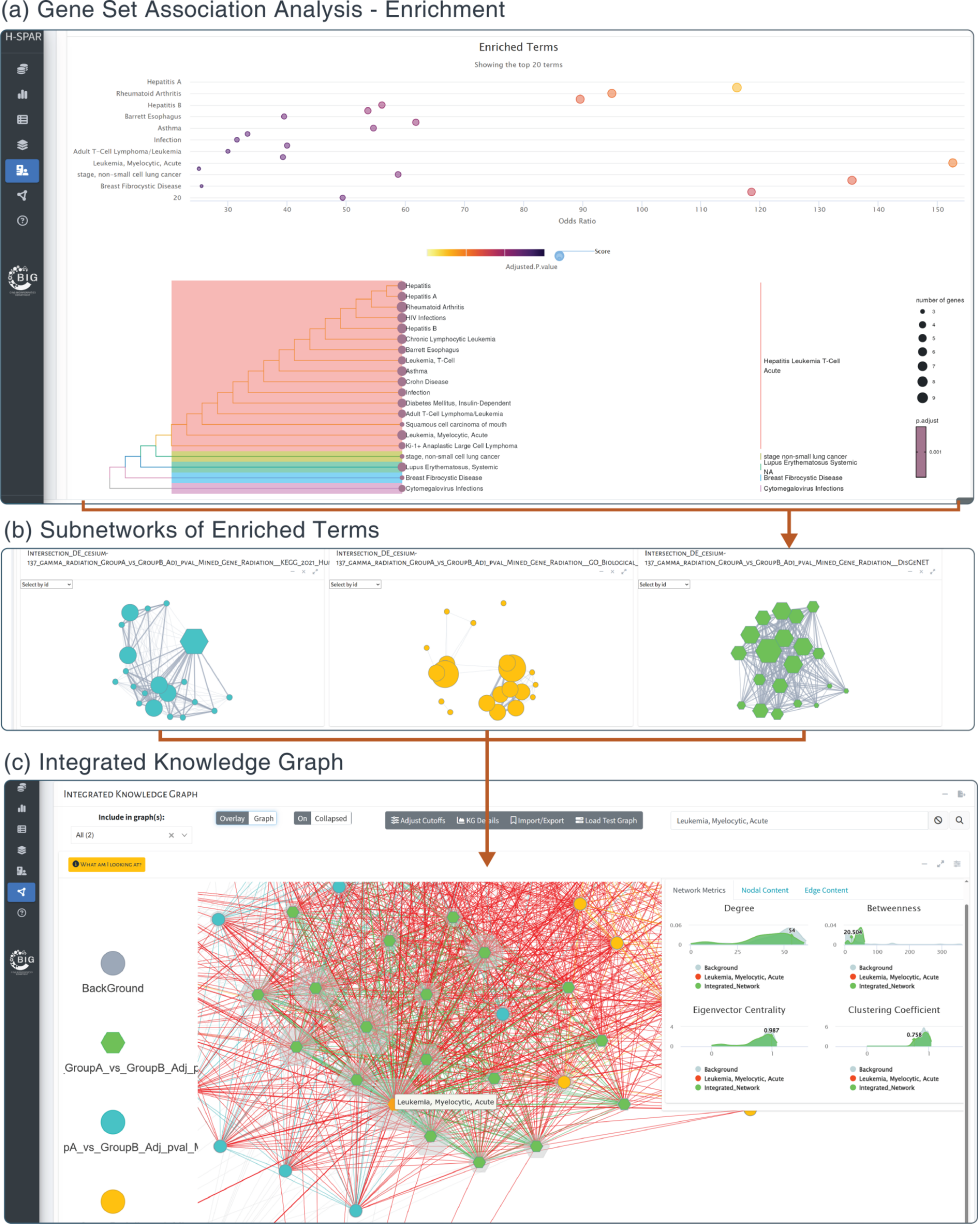
Application screenshots for enrichment and knowledge graph generation. (a) Results of the association analysis of specific gene sets are visualized as a dot plot of significantly enriched terms and a tree of their hierarchical clustering based on Jaccard’s similarity index. (b) Each selected enrichment set can be used to generate one subnetwork where nodes represent terms and edges represent common genes between them. The size of the node is proportional to the number of genes under the specific term and the weight of the edge is proportional to the number of common genes. (c) All subnetworks are unified under the knowledge graph where all enriched terms from any ontology or database are connected based on their gene-content commonality. Red edges denote connections between terms from different ontologies or databases (e.g. HPO and GO biological pathways), while other colours correspond to term connections within the same database.

To demonstrate H-SPAR DB’s capabilities, we used the tool to perform differential expression analysis in order to identify DEGs in response to cesium-137 gamma radiation, compared to non-irradiated samples. First, in the Differential Expression tab, we selected ‘Microarray’ as the data type. In Step 1, we specified ‘cesium-137 gamma radiation’ for Group A, and in Step 2, ‘Control: Non-irradiated’ for Group B. In Step 3, we entered the GeneLab dataset ID ‘OSD-129’ to fetch the corresponding experiment and ran the differential expression analysis directly through the platform. We identified 112 DEGs (adjusted *P*-value < .05, |log2FC| ≥ 1). Next, we searched the Mined Data for radiation-related genes and retrieved a set of 984 genes. By performing an intersection analysis, we found 10 common genes between our DEGs and the literature-derived dataset. To further explore the biological relevance of these genes, we conducted Association Analysis, including GO Biological Processes, Disease Association Analysis (DisGeNET), and KEGG pathways. We constructed a knowledge graph for each ontology, uncovering key molecular connections (Figs 1 and 2). Among the most highly connected diseases, Leukaemia and Hepatitis showed significant associations. KEGG pathway analysis revealed that Hepatitis B, MAPK signalling, and Pathways in Cancer had the highest degree of connectivity. Similarly, biological process analysis identified Apoptotic Process, Cellular Response to Light Stimulus, and Cellular Response to UV as highly interconnected within the knowledge graph. Moreover, the identified pathways and genes are well supported by previous research. Activation of MAPK signalling and apoptosis are central mechanisms in the cellular response to ionizing radiation and oxidative stress. Radiation-induced activation of ERK1/2 and JNK cascades promotes DNA-damage signalling and programmed cell death, consistent with our results [18, 19]. In addition, hepatic dysfunction and hematopoietic alterations, including leukemogenesis, are well-established consequences of radiation exposure [20–22]. These findings are consistent with known radiation-induced biological effects, demonstrating how H-SPAR DB efficiently uncovers relevant molecular mechanisms.

### Use case scenario 2: investigating age-related muscle responses in spaceflight

To explore age-related differences in muscle adaptation to spaceflight, we analysed transcriptomic data from young active and old sedentary muscle biopsies under microgravity conditions. Using the Differential Expression tab in H-SPAR DB, we selected age-specific factors and identified the OSD-684 RNA-seq dataset from NASA’s GeneLab. More specifically, in the Differential Expression tab, we selected ‘RNA’ as the data type. In Step 1, we specified ‘Space Flight’ for Group A, and in Step 2, ‘Ground Control’ for Group B. In Step 3, we entered the GeneLab dataset ID ‘OSD-684’ to fetch the corresponding experiment. Then we manually selected the different age groups to perform differential expression analysis separately for young (spaceflight vs. ground control) and old (sedentary spaceflight vs. control), revealing 357 and 771 DEGs, respectively (adjusted *P*-value < .05, |log2FC| ≥ 1).

Further operation analysis identified 220 DEGs common to both groups. To gain deeper biological insights, we conducted association analysis, performing disease association analysis using the DisGeNET database and pathway analysis via Reactome. Knowledge graph construction highlighted key molecular interactions, with degenerative polyarthritis emerging as a common highly interconnected disease for both age groups. Pathway analysis further revealed Nervous System Development as the most highly connected pathway in the old group, while Muscle Contraction was predominant in young samples. Notably, the Muscle Contraction pathway was also enriched in older individuals, underscoring its role in mediating muscle responses to nervous system signals, an essential process in understanding spaceflight-induced musculoskeletal adaptations [23]. These findings align closely with published studies describing the loss of neuromuscular connectivity and impaired muscle regeneration in aged individuals during microgravity exposure. Age-related reductions in satellite cell activity and mitochondrial dysfunction have been reported to exacerbate spaceflight-induced sarcopenia [24, 25]. Moreover, previous transcriptomic and proteomic studies have identified alterations in myosin heavy chain expression, calcium signalling, and extracellular matrix remodelling under simulated microgravity, supporting the biological pathways captured by H-SPAR DB [26, 27]. The convergence between our results and literature evidence confirms that H-SPAR DB reliably reproduces established molecular mechanisms of muscle atrophy and aging in spaceflight environments.

## Discussion

Spaceflight exposes astronauts to stressors like microgravity, radiation, and confinement, leading to muscle atrophy, bone loss, immune dysfunction, and increased cancer risk. Understanding these effects at the molecular level is vital for astronaut health, but analysing such space-related omics data remains challenging due to its complexity and dispersion across multiple sources, slowing discoveries. Aiming to help address the above, H-SPAR DB is designed to provide rapid, multi-level, and integrative analysis of the publicly available molecular data that have been generated under spaceflight-related or simulating conditions. It offers a versatile interactive interface allowing to approach crucial research questions such as expression profile differences induced by microgravity, radiation, and other stressors, derive the disease and phenotypic terms related to dysregulated gene sets, and connect these terms to biological pathways and candidate drugs. The exploratory analysis of the database conceptually can proceed in two modes: a) comparative: where two different stressors (e.g. type of radiation) or characteristics of the subjects (e.g. sex) can lead to dysregulation of different phenotypic or biological pathway terms or b) functional: where one can explore the molecular mechanisms that are connected and likely underlying a specific phenotypic or disease term.

A major challenge in space health research is the scarcity and limited nature of available datasets, which are often difficult and expensive to generate. Most data come from cell cultures, which may not fully reflect human physiology in space conditions. The restricted number of human datasets limits the robustness of statistical and enrichment analyses, and results should therefore be interpreted as hypothesis-generating rather than conclusive. Furthermore, enrichment-based approaches can introduce potential bias if not supported by experimental validation, emphasizing the need for cautious interpretation of pathway and disease associations. As space-related data grow, H-SPAR DB can consolidate and analyse this information, offering valuable insights into spaceflight’s molecular effects. Future updates and versions of H-SPAR DB will expand beyond gene-level analysis by incorporating systems-level modelling and integrating data from other species, providing a more comprehensive understanding of spaceflight-induced changes. Planned developments include the integration of multi-omics layers (transcriptomics, proteomics, and metabolomics) and cross-species homology mapping to facilitate comparative analyses between humans and model organisms used in space research. Furthermore, advanced artificial intelligence (AI) and machine learning (ML) methods, including Graph Neural Networks (GNNs), will be employed to exploit the knowledge graphs generated within H-SPAR DB for tasks such as link prediction (e.g. predict new associations) or node classification (e.g. predict the significance or the category of a node). These models will enable automated identification of molecular patterns, inference of missing associations, and prediction of key pathways or candidate drugs across stressor conditions. The integration of AI/ML approaches aims to enhance hypothesis generation and accelerate discovery in space health and astrobiology.

To further assess the reliability and complementarity of H-SPAR DB, we compared its functionalities with GeneLab database, which serves as the main public repository of spaceflight-related omics data. The comparison shows that while both platforms offer visualization and association analyses, they differ in scope and analytical depth. GeneLab primarily focuses on the standardized processing of raw omics data and multi-omics integration across species, whereas H-SPAR DB incorporates manual curation from the literature and provides advanced downstream analyses, including set operation analyses, network analysis metrics, and knowledge graph construction. These additional layers enable users to interpret molecular patterns within broader biological and phenotypic contexts. This benchmarking demonstrates that H-SPAR DB complements GeneLab by extending its findings through curated, interpretable, and integrative analyses designed to accelerate hypothesis generation in space biology.

Stressing the fact that the results are not clinically applicable, the database can facilitate the prioritization and design of further functional studies to decipher the exact pathogenicity mechanisms of space-related conditions and candidate drug targets. To our knowledge, no other database has specifically integrated and analysed diverse biological data with a focus on human health in spaceflight. H-SPAR DB offers significant potential for astrobiology and space medicine researchers, providing valuable insights into the molecular effects of space environments and supporting strategies for astronaut health, mission success, and further impact for health-related studies on earth.

## Supplementary Material

baaf083_Supplemental_File

## Data Availability

H-SPAR DB can be accessed through https://bioinformatics.cing.ac.cy/H-SPARDB/. This website is free and open to all users and there is no login requirement.
