# Supplementary material for: H-SPAR DB: human spaceflight platform for analysis and research—an integrative omics database for space health
Source: Database (Oxford). 2026 Jan 15;2026:baaf083. doi: 10.1093/database/baaf083 (PMC12805116; doi:10.1093/database/baaf083)
Supplement: baaf083_Supplemental_File [file baaf083_supplemental_file.pdf]

H-SPAR DB: Human Spaceflight Platform for Analysis and Research – An Integrative Omics Database for Space Health

Marios Tomazou[1†], Marilena M. Bourdakou[1†], Eleni Nicolaidou[1], Grigoris Georgiou[1], Kyriaki Savva[1], Efi Athieniti[1], Styliana Menelaou[1], Sotiroula Afxenti[1] and George M. Spyrou[1*]

[1]Bioinformatics Department, The Cyprus Institute of Neurology and Genetics, 2371 Nicosia, Cyprus

* Correspondence: Tel: +357 - 22 392852 Fax: +357 22 358 238 Email: georges@cing.ac.cy

Supplementary Materials

Table S1: PubMed search queries for space-related biological studies

Pubmed Queries for Mined Data	Number_Of_Publications
(protein*[tiab] OR proteomic*[tiab]) AND spaceflight[ti]	215
(gene*[tiab] OR genomic*[tiab]) AND spaceflight[ti]	436
(microRNA*[tiab] OR miRNA*[tiab]) AND spaceflight[ti]	21
(variant*[tiab] OR mutation*[tiab]) AND spaceflight[ti]	37
(metabolite*[tiab] OR metabolomic*[tiab]) AND spaceflight[ti]	43
(lipid*[tiab] OR lipidomic*[tiab]) AND spaceflight[ti]	30
(gene[tiab] OR genomic*[tiab]) AND "solar wind"[tiab]	No
(genes[tiab] OR genomic*[tiab]) AND "solar wind"[tiab]	No
(protein*[tiab] OR proteomic*[tiab]) AND "solar wind"[tiab]	2
(microRNA*[tiab] OR miRNA*[tiab]) AND "solar wind"[tiab]	No
(variant*[tiab] OR mutation*[tiab]) AND "solar wind"[tiab]	1
(metabolite*[tiab] OR metabolomic*[tiab]) AND "solar wind"[tiab]	No
(lipid*[tiab] OR lipidomic*[tiab]) AND "solar wind"[tiab]	No
(gene[tiab] OR genomic*[tiab]) AND "solar particle"[tiab]	12
(genes[tiab] OR genomic*[tiab]) AND "solar particle"[tiab]	11
(protein*[tiab] OR proteomic*[tiab]) AND "solar particle"[tiab]	12
(microRNA*[tiab] OR miRNA*[tiab]) AND "solar particle"[tiab]	3
(variant*[tiab] OR mutation*[tiab]) AND "solar particle"[tiab]	4
(metabolite*[tiab] OR metabolomic*[tiab]) AND "solar particle"[tiab]	3
(lipid*[tiab] OR lipidomic*[tiab]) AND "solar particle"[tiab]	1
(gene[tiab] OR genomic*[tiab]) AND "solar weather*"[tiab]	No
(genes[tiab] OR genomic*[tiab]) AND "solar weather*"[tiab]	No
(protein*[tiab] OR proteomic*[tiab]) AND "solar weather*"[tiab]	No
(microRNA*[tiab] OR miRNA*[tiab]) AND "solar weather*"[tiab]	No
(variant*[tiab] OR mutation*[tiab]) AND "solar weather*"[tiab]	No
(metabolite*[tiab] OR metabolomic*[tiab]) AND "solar weather*"[tiab]	No
(lipid*[tiab] OR lipidomic*[tiab]) AND "solar weather*"[tiab]	No
(gene[tiab] OR genomic*[tiab]) AND "solar storm"[tiab]	1
(genes[tiab] OR genomic*[tiab]) AND "solar storm"[tiab]	No
(protein*[tiab] OR proteomic*[tiab]) AND "solar storm"[tiab]	No
(microRNA*[tiab] OR miRNA*[tiab]) AND "solar storm"[tiab]	No
(variant*[tiab] OR mutation*[tiab]) AND "solar storm"[tiab]	No
(metabolite*[tiab] OR metabolomic*[tiab]) AND "solar storm"[tiab]	No

(lipid*[tiab] OR lipidomic*[tiab]) AND "solar storm"[tiab]	No
(gene[tiab] OR genomic*[tiab]) AND "solar flare*"[tiab]	No
(genes[tiab] OR genomic*[tiab]) AND "solar flare*"[tiab]	No
(protein*[tiab] OR proteomic*[tiab]) AND "solar flare*"[tiab]	3
(microRNA*[tiab] OR miRNA*[tiab]) AND "solar flare*"[tiab]	No
(variant*[tiab] OR mutation*[tiab]) AND "solar flare*"[tiab]	6
(metabolite*[tiab] OR metabolomic*[tiab]) AND "solar flare*"[tiab]	No
(lipid*[tiab] OR lipidomic*[tiab]) AND "solar flare*"[tiab]	2
(gene[tiab] OR genomic*[tiab]) AND "space radiation"[ti]	26
(genes[tiab] OR genomic*[tiab]) AND "space radiation"[ti]	18
(protein*[tiab] OR proteomic*[tiab]) AND "space radiation"[ti]	25
(microRNA*[tiab] OR miRNA*[tiab]) AND "space radiation"[ti]	2
(variant*[tiab] OR mutation*[tiab]) AND "space radiation"[ti]	15
(metabolite*[tiab] OR metabolomic*[tiab]) AND "space radiation"[ti]	3
(lipid*[tiab] OR lipidomic*[tiab]) AND "space radiation"[ti]	3
(gene[tiab] OR genomic*[tiab]) AND "microgravity"[ti]	351
(genes[tiab] OR genomic*[tiab]) AND "microgravity"[ti]	318
(protein*[tiab] OR proteomic*[tiab]) AND "microgravity"[ti]	592
(microRNA*[tiab] OR miRNA*[tiab]) AND "microgravity"[ti]	42
(variant*[tiab] OR mutation*[tiab]) AND "microgravity"[ti]	30
(metabolite*[tiab] OR metabolomic*[tiab]) AND "microgravity"[ti]	48
(lipid*[tiab] OR lipidomic*[tiab]) AND "microgravity"[ti]	54
(gene[tiab] OR genomic*[tiab]) AND "hypergravity"[ti]	61
(genes[tiab] OR genomic*[tiab]) AND "hypergravity"[ti]	51
(protein*[tiab] OR proteomic*[tiab]) AND "hypergravity"[ti]	82
(microRNA*[tiab] OR miRNA*[tiab]) AND "hypergravity"[ti]	1
(variant*[tiab] OR mutation*[tiab]) AND "hypergravity"[ti]	4
(metabolite*[tiab] OR metabolomic*[tiab]) AND "hypergravity"[ti]	No
(lipid*[tiab] OR lipidomic*[tiab]) AND "hypergravity"[ti]	15
(organ-on-chip[tiab]) AND "hypergravity"[ti]	No
("organ on chip"[tiab]) AND "hypergravity"[ti]	No
(organ-on-a-chip[tiab]) AND "hypergravity"[ti]	No
("tissue chip"[tiab]) AND "hypergravity"[ti]	No
(organ-on-chip[tiab]) AND "microgravity"[ti]	1
("organ on chip"[tiab]) AND "microgravity"[ti]	1
(organ-on-a-chip[tiab]) AND "microgravity"[ti]	2
("tissue chip"[tiab]) AND "microgravity"[ti]	2
(organ-on-chip[tiab]) AND "space radiation"[ti]	No
("organ on chip"[tiab]) AND "space radiation"[ti]	No
(organ-on-a-chip[tiab]) AND "space radiation"[ti]	1
("tissue chip"[tiab]) AND "space radiation"[ti]	No
(organ-on-chip[tiab]) AND "spaceflight"[ti]	No
("organ on chip"[tiab]) AND "spaceflight"[ti]	No
(organ-on-a-chip[tiab]) AND "spaceflight"[ti]	1
("tissue chip"[tiab]) AND "spaceflight"[ti]	1

Table S2: GeneLab transcriptomic datasets

	OSD	Title	Assay	Organism	Factor	Measurement	Platform
Microarrays	OSD-124	Effect of electromagnetic fields on the chondrogenic differentiation under microgravity conditions	DNA microarray	Homo sapiens	electromagnetic fields	transcription profiling	Affymetrix
	OSD-125	mRNA expression profile in DLD-1 and MOLT-4 cancer cell lines cultured under Microgravity	DNA microarray	Homo sapiens	cell line, microgravity simulation	transcription profiling	Affymetrix
	OSD-129	Gene expression profiling of PBL in response to ionising radiation and modeled microgravity	DNA microarray	Homo sapiens	ionizing radiation, simulated microgravity	transcription profiling	Agilent
	OSD-13	T Cell Activation in Microgravity Compared to 1g (Earth's) Gravity	DNA microarray	Homo sapiens	microgravity simulation, spaceflight, treatment	transcription profiling	Affymetrix
	OSD-151	Bystander response to 2.5 Gy of protons in a human 3-dimensional skin model in 16 h after exposure	DNA microarray	Homo sapiens	ionizing radiation	transcription profiling	Agilent
	OSD-152	Transcription profiling of human peripheral blood to development gene expression signatures for practical radiation biodosimetry	DNA microarray	Homo Sapiens	absorbed radiation dose, ionizing radiation, time of sample collection after treatment	transcription profiling	Agilent
	OSD-154	Low dose ionizing radiation treated lymphoblastoid cells	DNA microarray	Homo sapiens	absorbed radiation dose, cell line ,ionizing radiation	transcription profiling	Affymetrix
	OSD-172	Dynamic gene expression response to altered gravity in human T cells (parabolic flight)	microarray	Homo sapiens	microgravity	transcription profiling	Affymetrix
	OSD-178	IMR90 radiation bystander time-course experiment 0.5Gy alpha particle	DNA microarray	Homo sapiens	absorbed radiation dose, ionizing radiation, time	transcription profiling	Agilent
	OSD-182	Human skin fibroblast mitochondrial depletion, 0.5 Gy alpha-particle	DNA microarray	Homo sapiens	dose, ionizing radiation, mitochondrial status	transcription profiling	Agilent
	OSD-188	Dynamic gene expression response to altered gravity in human T cells (sounding rocket flight)	microarray	Homo sapiens	altered gravity	transcription profiling	Affymetrix
	OSD-367	Bystander response to 0.5 Gy of alpha-particles in a human 3-dimensional skin model in 16h after exposure to ionizing radiation	DNA microarray	Homo sapiens	ionizing radiation, radiation distance	transcription profiling	Agilent
	OSD-368	Biological response to low dose of alpha-	DNA microarray	Homo sapiens	ionizing radiation, time	transcription profiling	Agilent

		particles in a human 3-dimensional skin model, in 1 and 16h after exposure to ionizing radiation.			of sample collection after treatment		
	OSD-369	Bystander responses to 0.5Gy of alpha-particles in a human 3-dimensional skin model in 4h after exposure to ionizing radiation	DNA microarray	Homo sapiens	ionizing radiation	transcription profiling	Agilent
	OSD-370	Insulin resistance induced by physical inactivity is associated with multiple transcriptional changes in skeletal muscle in young men	DNA microarray	Homo sapiens	bed rest, time, treatment	transcription profiling	Agilent
	OSD-52	Expression data from SPHINX (SPaceflight of Huvec: an INtegrated eXperiment)	DNA microarray	Homo sapiens	spaceflight	transcription profiling	Affymetrix
	OSD-542	Effects of 14 days of confinement on blood gene expression profiles in men	DNA microarray	Homo sapiens	time	transcription profiling	Agilent
	OSD-546	SCD – Stem Cell Differentiation Toward Osteoblast Onboard the International Space Station	DNA microarray	Homo sapiens	spaceflight, treatment	transcription profiling	Affymetrix
	OSD-71	Immediate Transcriptional Changes in Response to High Dose Radiation Exposure	DNA microarray	Homo sapiens	absorbed radiation dose, ionizing radiation	transcription profiling	Affymetrix
	OSD-78	Transcription profiling of human MCF10A cells subjected to ionizing radiation and treatment with transforming growth factor beta-1	DNA microarray	Homo sapiens	ionizing radiation, treatment	transcription profiling	Affymetrix
	OSD-92	Response of the EPI-200 human 3-D skin model to high and low doses of protons	DNA microarray	Homo sapiens	radiation, timepoint	transcription profiling	Agilent
**RNA-seq**	OSD-577	3D Human Umbilical Vein Endothelial Cells (HUVECs) irradiated with GCR simulated irradiation with miRNA based countermeasures	RNA Sequencing (RNA-Seq)	Homo sapiens	ionizing radiation, tissue model, treatment	transcription profiling	Illumina
	OSD-91	A study of gene expression influenced by simulated microgravity in human lymphoblastoid cells	RNA Sequencing (RNA-Seq)	Homo sapiens	microgravity simulation	transcription profiling	Illumina
	OSD-258	Effects of Spaceflight on Human Induced Pluripotent Stem Cell-Derived Cardiomyocyte Structure and Function	RNA Sequencing (RNA-Seq)	Homo sapiens	spaceflight, time	transcription profiling	Illumina HiSeq 2000

OSD-323	Evaluating the effect of spaceflight on the host-pathogen interaction between human intestinal epithelial cells and Salmonella Typhimurium	RNA Sequencing (RNA-Seq)	Homo sapiens	infection, spaceflight	transcription profiling	Illumina
OSD-127	Global gene expression profiles of cardiac progenitors differentiated from human pluripotent stem cells in 3D culture under simulated microgravity	RNA Sequencing (RNA-Seq)	Homo sapiens	microgravity simulation	transcription profiling	Illumina HiSeq1000
OSD-431	Microgravity and space radiation exert either opposite or synergic effects on single molecular pathways	RNA Sequencing (RNA-Seq)	Homo sapiens	altered gravity, spaceflight	transcription profiling	Illumina
OSD-540	Ten-day human unilateral lower limb suspension and active recovery in young healthy men	RNA Sequencing (RNA-Seq)	Homo sapiens	hind limb unloading	transcription profiling	Illumina
OSD-539	Transcriptomic response of bioengineered human cartilage to parabolic flight microgravity is sex-dependent	RNA Sequencing (RNA-Seq)	Homo sapiens	sex, treatment	transcription profiling	Illumina
OSD-684	Human skeletal muscle tissue chip autonomous payload reveals changes in fiber type and metabolic gene expression due to spaceflight.	RNA Sequencing (RNA-Seq)	Homo sapiens	spaceflight, time	transcription profiling	Illumina
OSD-516	Modeling cellular responses to serum and vitamin D in microgravity using a human kidney microphysiological system	RNA Sequencing (RNA-Seq)	Homo sapiens	spaceflight, treatment, sex	transcription profiling	Illumina

Table S3: Selected databases from Enrichr R package

Library	URL	Number of Terms	Number of Genes	Category
BioCarta_2016	http://cgap.nci.nih.gov/Pathways/BioCarta_Pathways	237	1348	Pathways
ClinVar_2019	https://www.ncbi.nlm.nih.gov/clinvar/	182	1397	Diseases
COVID-19_Related_Gene_Sets_2021	https://maayanlab.cloud/covid19/	478	16853	Diseases
dbGaP	http://www.ncbi.nlm.nih.gov/gap	345	5613	Diseases
DGIdb_Drug_Targets_2024	https://dgidb.org/	659	2513	Drugs
DisGeNET	https://www.disgenet.org	9828	17464	Diseases
DSigDB	http://tanlab.ucdenver.edu/DSigDB/DSigDBv1.0/	4026	19513	Diseases
GO_Biological_Process_2025	http://www.geneontology.org/	5343	14698	Ontologies

GO_Cellular_Component_2025	http://www.geneontology.org/	468	10972	Ontologies
GO_Molecular_Function_2025	http://www.geneontology.org/	1174	12126	Ontologies
GWAS_Catalog_2023	https://www.ebi.ac.uk/gwas	5271	18290	Diseases
Human_Phenotype_Ontology	http://www.human-phenotype-ontology.org/	1779	3096	Diseases
IDG_Drug_Targets_2022	https://drugcentral.org/	888	1552	Drugs
KEGG_2021_Human	https://www.kegg.jp/	320	8078	Pathways
miRTarBase_2017	http://mirtarbase.mbc.nctu.edu.tw/	3240	14893	MicroRNAs
OMIM_Disease	http://www.omim.org/downloads	90	1759	Diseases
Reactome_Pathways_2024	https://reactome.org/download-data	2105	11671	Pathways
SynGO_2024	https://www.syngoportal.org/	134	1555	Ontologies
TargetScan_microRNA_2017	http://www.targetscan.org/	683	17598	MicroRNAs
Tissue_Protein_Expression_from_Human_Proteome_Map	http://www.humanproteomemap.org/index.php	30	6454	Diseases
VirusMINT	http://mint.bio.uniroma2.it/download.html	85	851	Diseases
WikiPathways_2024_Human	https://www.wikipathways.org/	829	8281	Pathways
